# Perioperative therapy for locally advanced gastroesophageal cancer: current controversies and consensus of care

**DOI:** 10.1186/1756-8722-6-66

**Published:** 2013-09-05

**Authors:** Amikar Sehdev, Daniel VT Catenacci

**Affiliations:** 1 Department of Medicine, Section of Hematology Oncology, University of Chicago, 5841 S. Maryland Avenue, MC 2115, Chicago, IL 60637, USA

**Keywords:** Gastric adenocarcinoma, Stomach cancer, Esophageal adenocarcinoma, Esophagogastric (gastroesophageal) junction cancer, Chemotherapy, Chemoradiotherapy, Multi-disciplinary care

## Abstract

Gastroesophageal cancer (GEC) remains a challenging problem in oncology. Anatomically, GEC is comprised of distal gastric adenocarcinoma (GC), classically associated with *Helicobacter Pylori,* while proximal esophagogastric adenocarcinoma (EGJ AC) has increased significantly in incidence over the past years. Despite contrasting etiologies, histologies, and molecular phenotypes of distal and proximal GEC, in many cases perioperative (and metastatic) treatment strategies converge to similar approaches. For patients undergoing curative intent surgery, advances in perioperative chemotherapy and/or chemoradiotherapy, either before and/or after surgery, have demonstrated improved survivals compared to surgery alone. This review focuses on how the ‘boundary’ of the Z-line and/or the anatomical distinction of ‘proximal’ (EGJ) vs. ‘distal’ (GC) cancer has led to diverse inclusion/exclusion criteria for clinical trial enrollment, embodying various combinations of chemotherapy and radiation before and/or after surgery. Supporting evidence of each of these approaches consequently has led to a number of varying practices by geographical region and Institution/Physician, based on differing experience, preference, and clinical circumstance. Adequate direct comparison of these approaches is lacking currently, but data from a number of concerted efforts should be available in the next years to further direct best standards of care. Introduction of biologically targeted agents, namely anti-angiogenics and anti-HER family therapeutics are being evaluated to determine whether further therapeutic gains can be realized over classic cytotoxic chemotherapy alone (with/without radiotherapy). To date, novel molecularly targeted agents have yet to demonstrate benefit in this setting. In the following comprehensive review we will address the intricacies of perioperative treatment of locally advanced GEC, with focus on clinical trials supporting the diverse set of perioperative multidisciplinary approaches.

## Current curative standards and controversies

Gastroesophageal adenocarcinoma (GEC) is a complex disease which can be broadly classified into proximal esophagogastric junction (EGJ) and distal gastric cancer (GC) [1]. At present, surgery is the sole curative option for operable GEC. Endoscopic mucosal resection (EMR) is also a possible local therapy for well-differentiated, non-depressed tumors that are less than 2 cm in size and invading only up to superficial muscularis (T1a) [2]. EMR for T1aN0 disease is associated with 5-year survival comparable to surgery and has relatively low mortality and morbidity [3]. EMR alone is currently still controversial for T1b (sm1), with reports of 1-3% LN involvement, while sm2 and sm3 have 10-30% LN involvement [3]. Additionally, EMR can be curative for poorly differentiated intramucosal lesions less than 1 cm, and in non-poorly differentiated or non-ulcerated tumors less than 2 cm in size [4].

For more advanced disease, the type of surgical approach and extent of surgery depends upon the anatomical location, extent, and TNM stage of the tumor, and is beyond the scope of this review [3]. For the most part, there is consensus regarding surgical approach for Siewert type I (considered esophageal cancers), treated by either an en bloc transthoracic or transhiatal esophagectomy with two-field lymphadenectomy. For type III EGJ tumors (gastric cardia), a total gastrectomy via laparotomy and D2 lymphadenectomy without routine distal splenopancreatectomy is recommended. For Siewert type II EGJ tumors, either of these procedures are accepted approaches [3,5]. Although there is some controversy, proximal stomach tumors (including gastric cardia) require either a total gastrectomy or proximal gastrectomy with resection of 5 to 10 cm of esophagus. Tumors of the middle third/fundus of the stomach usually require a total gastrectomy, however tumors of the distal third of the stomach can undergo radical subtotal (75-85%) gastrectomy [6]. Most trials have demonstrated that achieving a R0 resection is critical and is prognostic of improved 5-year survival for both EGJ and GC, in contrast to R1/R2 resection (microscopic/macroscopic tumor) [7].

With respect to D1 versus D2 dissection, a recent metaanalysis evaluating 5 RCTs, involving 1642 patients with GC enrolled from 1982 to 2005, revealed a higher operative mortality associated with D2 dissections in the earlier trials, while recent trials have similar rates of mortality between D2 and D1 lymphadenectomy [8]. A trend of improved survival existed among D2 patients who did not undergo resection of the spleen or pancreas, as well as for patients with T3/T4 cancers. Given that D2 resections may improve the accuracy of locoregional staging and may improve survival when avoiding routine distal pancreatectomy and splenectomy, most agree that this modified D2 approach is appropriate and has been accepted as standard of care in the recent NCCN guidelines [9]. The D1 versus D2 variable plays a significant role when comparing results across various trials (reported and ongoing) that will be discussed below.

**Figure 1:**
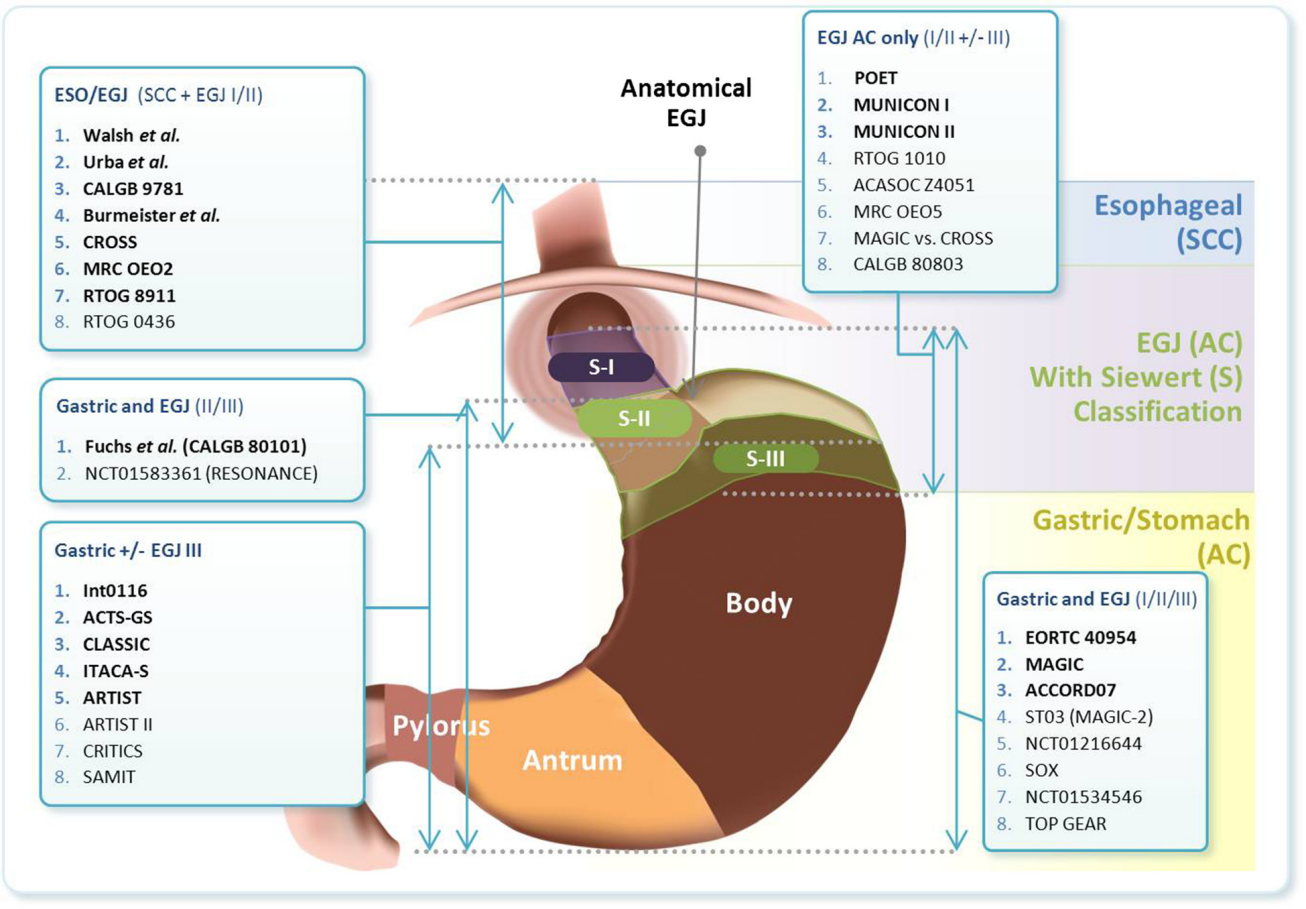
**Completed and ongoing perioperative gastroesophageal adenocarcinoma clinical trials, demonstrating enrollment criteria by tumor location.** See text and tables for trial references. Abbreviations: SCC, squamous cell carcinoma; EGJ, esophagogastric junction; AC, adenocarcinoma. Bolded: clinical trials with results reported; Not Bolded: clinical trials that are ongoing.

The complexity and heterogeneity of GEC, in terms of patient ethnicity [10], as well as anatomical, histological, and molecular subsets, has resulted in a number of categorizations, perioperative treatment strategies, and surgical approaches [1,11]. Specifically, the anatomical distinction of ‘proximal’ (EGJ) versus ‘distal’ (GC) cancer has led to diverse inclusion/exclusion criteria for clinical trial enrollment, embodying various combinations of chemotherapy and radiation before and/or after surgery (Figure 1). Supporting evidence of each of these approaches consequently has led to a number of different practices by geographical region and Institution/Physician, based on varying experience and preference, and discussed in further detail below.

## Modalities of perioperative treatment

Surgery is the required modality of curative intent treatment of locally advanced GEC. Occult peritoneal disease as the sole site of dissemination reportedly occurs in approximately 20-25% of GEC patients, according to several large retrospective analyses [1]. Perioperative debulking, hyperthermic intraperitoneal chemotherapy (HIPEC), and other novel immunological approaches have been evaluated as part of the curative intent strategy, mostly in Asia, recently reviewed elsewhere [1]. A number of clinical trials have established various perioperative treatment options that further improve mOS compared to surgery alone, including i) neoadjuvant chemoradiation (CRT→**S**), ii) adjuvant CRT (**S**→CRT), iii) neoadjuvant chemotherapy (C→**S**), iv) adjuvant chemotherapy (**S**→C), v) perioperative chemotherapy (sandwich approach) (C→**S**→C), and vi) induction chemotherapy followed by neoadjuvant CRT (C→CRT→**S**). The following is a summary of phase III trials supporting each of these strategies (Table 1; Figure 1).

**Table 1:** Phase III* clinical trials evaluating perioperative therapy for gastroesophageal adenocarcinoma

**Study**	**Inclusion**	**Treatment and number of patients**	**AC/SCC (%)**	**ITT R0**	**pCR (%)**	**DR (%)**	**LR (%)**	**Location (%)**				**3-year OS (%)**
	**AJCC 6**^**th**^**Ed.**							**EGJ Seiwert**			**GC**	
	**T/N**							**I**	**II**	**III**		
**i. Neoadjuvant CRT versus surgery alone**												
Walsh et al. [12], 1996	Locally advanced RT: 40 Gy	A: CRT (PF) → S: 58	100	NR	A: 25	NR	NR	Esophagus (16 middle 1/3^rd^; 51 lower 1/3^rd^; 34 cardia)			0	A: 32
Irish Trial		B: S: 55			B: 0							B: 6
												p = 0.01
Burmeister et al. [13], 2005	T1-3, N0-1	A: CRT (PF) → S: 128	62/37	A: 80	A: 12	A: 45	A: 11	Esophagus (21, upper and middle 1/3; 79 distal 1/3)			0	A: 32
	RT: 35 Gy	B: S: 128		B: 59	B: 0	B: 41	B: 14					B: 29
												p = 0.83
Tepper et al. [14], 2008	T1-3, Nx	A: CRT (PF) → S: 30	75/25	NR	A: 40	A: 27	A: 3	Thoracic esophagus & EGJ			0	A: 39 (5-year)
CALGB 9781	RT: 50.4 Gy	B: S: 26			B: 0	B: 38	B: 11					B: 16
												p < 0.002
Urba et al. [15], 2001	Locally advanced	A: CRT (PFV) → S: 50		NR	A: 28	A: 65	A: 19				0	A: 30
	RT: 45 Gy	B: S: 50	75/25		B: 0	B: 60	B: 42	Esophagus (8 upper 1/3rd; 92 mid and distal 1/3rd)				B: 16
												p = 0.15
van Hagen et al. [18], 2012	T1N1 or T2-T3N0-1	A: CRT (TP) → S: 175	75/25	92 vs. 69	A: 29	A: NR	A: NR	49	26	0	0	A: 58
‘CROSS’ Trial	Ib-IIIb	B: S: 188			AC: 23%	B: NR	B: NR					B: 44
	RT: 41.4 Gy				B: 0							HR 0.65 (0.49-0.87)
												p = 0.003
												Subgroup analysis for AC only: HR 0.74 (0.53-1.02)
												p = 0.07
**ii. Adjuvant CRT versus surgery alone**												
Macdonald et al. [19], 2001	T1 – 4 N + Ib – IV	A: S: → CRT 281	100/0	NA	NA	A: 33	A: 19	0	0	20	80	A: 50
Intergroup 0116	RT: 45 Gy	C(5FU) – CRT(5FU) – C(5FU)				B: 18	B: 29					B: 41
		B: S: 275										HR 0.76 (0.62-0.96)
												p = 0.005
**iii. Neoadjuvant chemotherapy versus surgery alone**												
MRC OEO-2 [27], 2002	Resectable cancer	A: C (CF) → S: 400	66/31	A: 60	NR	A: 12	A: 8	64		10	0	A: 43 (2 year)
		B: S: 402		B: 54		B: 10	B: 8	(26 SCC)				B: 34
												HR 0.78 (0.67-0.93)
												p = 0.004
Schuhmacher et al. [28], 2010	T3 – T4, N0 – N + (Locally Advanced)	A: C (CF) → S: 72	100/0	A: 84	A: 7	NR	NR	53			47	A: 72.7 (2 year)
EORTC 40954		B: S: 72		B: 72	B: 0							B: 69.9
												p = 0.46
**iv. Adjuvant chemotherapy versus surgery alone**												
Sakuramoto et al. [31], 2007	T1 – 4 N + II (excluding T1) – IIIB	A: S → C (S1) 529	100/0	NA	NA	A: 21.4	A: 2.8	0	0	0	100	A: 80
ACTS-GS Trial		B: S: 530				B: 27.1	B: 1.3					B: 70
												HR 0.68 (0.5-0.87)
												p = 0.003
Bang et al. [34], 2012	T – 4 N + or II-IIIB	A: S → C (CapeOx): 520	100/0	NA	NA	A: 18	A: 4	0	0	2.5	97.5	A: 74 (3-year DFS)
‘CLASSIC’ Trial		B: S: 515				B: 26	B: 8					B: 59
												HR 0.56 (0.44-0.72)
												p < 0.0001
**v. Perioperative chemotherapy versus surgery alone**												
Kelsen et al. [36,37], 1998, 2007	I-III	A: C (CF) → S → CF 233	51/44	A: 62	A: 2.5	A: 49	A: 19	51			0	A: 26
RTOG 8911	Any N +	B: S: 234		B: 59	B: 0	B: 51	B: 21					B: 23
												p = 0.53
Cunningham et al. [38]*,* 2006	T1-4 N + or II-IV	A: C (ECF) → S → C (ECF): 250	100/0	A: 69	4	A: 24	A: 14	14	12		74	A: 36 (5-year)
‘MAGIC Trial’		B: S: 253		B: 66		B: 37	B: 21					B: 23
												HR 0.75 (0.6-0.9)
												p = 0.009
Ychou et al. [39], 2011	T1-4 N + or II-IV	A: C (CF) → S → C (CF): 113	100/0	A: 84	3	A: 30	A: 24	11	64		25	A: 38 (5-year)
FFCD9703 Trial						B: 38	B: 26					B: 24
ACCORD 07		B: S: 111		B: 74								5-yr HR 0.69 (0.5-0.95); p = 0.02
**vi. Comparison of post-operative chemotherapy regimens**												
Bajetta et al. [43], 2012	N + or N0 with T2b-4	A: S → C (CPT-11 + 5-FU/LV – TXT + CDDP): 562	100/0	NA	NA	NR	NR	0	0	15	85	No significant difference
Abstract NCT01640782 ITACA-S Trial		B: S → C (5-FU/LV): 538										HR: 1.00 (0.83-1.20); p = 0.98
Kobayashi Yoshida et al. [44], 2012	T3-4, N0-2	A: S → C (UFT): 359	100/0	NA	NR	NR	NR	None			100	No significant difference in DFS between C + D and A + B
‘SAMIT’ Trial		B: S → C (S1): 364										HR: 0.92 (0.80-1.07); p = 0.273
		C: S → C (T – > UFT): 355										
		D: S → C (T – > S1): 355										
**vii. Adjuvant CRT comparing peri-RT chemotherapy regimens**												
Fuchs et al. [46], 2011	Locally Advanced	A: S → C (5FU/LV) → CRT (5FU) → C (5FU/LV): 280	100/0	NR	NR	NR	NR	None			100	A: 50
CALGB 80101		B: S → C (ECF) → CRT (5FU) → C (ECF): 266										B: 52
NCT00052910												HR 1.03 (0.80-1.34) p = 0.80
**viii. PET directed therapy**												
Lordick et al. [50], 2007	cT3/4 Locally advanced	C (CF) → Assess for metabolic response	100/0	NA	A: 58	NR	NR	68	32	0	0	A: Not reached
Phase II Municon trial		A: Response → C → S: 54			B: 0							B: 25.8 (2.3 years median follow up)
		B: No-Response → S: 56			(Major Histologic Response)							HR = 2.13 (1.14-3.99)
												P = 0.015
zum Buschenfelde et al. [52], 2011	cT3/4 Locally advanced	C (CF) → Assess for metabolic response	100/0	NA	A: 36	A: 30	A: 9	69	31	0	0	A: 71
Phase II Municon II		A: Response → C → S: 33			B: 26	B: 48	B: 17					B 42
		B: No-Response → CRT → S: 23			(Major Histologic Response)							HR = 1.9 (0.87-4.24)
												P = 0.10
**ix. Induction chemotherapy followed by CRT versus chemotherapy or CRT alone**												
Stahl et al. [57], 2009,	T3/4, Nx	A: C (PLF) → CRT (PE) → S: 60	100/0	A: 72	A: 16	A: 23	NR	54	46		0	A: 47.4
‘POET’ Trial		B: C (PLF) → S: 59		B: 70	B: 2	B: 41						B: 27.7
												HR 0.67 (0.41-1.07)
												p = 0.07
Alberts et al. [58], 2013	T3-4, N0 or Tany, N(+)	A: C (DOX) → CRT (5Fu + Ox): 21	100/0	NA	A: 33	NR	NR	40			0	NR
N0849 Trial		B: CRT alone: 21			B: 48			(SCC 55)				
					P = 0.53							
**x. Adjuvant chemotherapy versus CRT**												
Lee et al. [26], 2012	II-IV	A: S → C (XP): 228	100/0	NA	NA	A: 25	A: 8.3	0	0	5	95	A: 74.2 (3-year DFS)
‘ARTIST’ Trial	Any N+	B: S → C (XP) → CRT (X) → C (XP): 230				B: 20	B: 4.8					B: 78.2
							p = 0.35					p = 0.086

* unless otherwise indicated.

*Abbreviations:AC* adenocarcinoma; *SCC* squamous cell carcinoma; *ITT* intention to treat; *pCR* pathological complete response; *DR* distant recurrence; *LR* local recurrence; *EGJ* esophagogastric junction; *GC* distal gastric cancer; *OS* overall survival; *RT* radiation therapy; *CRT* chemoradiotherapy; *PF* cisplatin and 5-flourouracil; *NR* not reported; *PFV* cisplatin, 5-flourouracil and vinblastine; *TP* carboplatin and paclitaxel; *5FU/L* 5-flouroacil and leucovorin; *NA* not applicable; *HR* hazard ratio; *C* chemotherapy alone; *CF* cisplatin and 5-flourouracil; *CapeOx* capecitabine and oxaliplatin; *XP* capecitabine and cisplatin; *ECF* epirubicin, cisplatin and 5-flourouracil; *PLF* cisplatin, leucovorin and 5-flourouracil; *PE* cisplatin and etoposide; *TXT* docetaxel; *CPT-11* Irinotecan; *CDDP* cisplatin.

## Comparisons to surgery alone

### i. Neoadjuvant chemoradiotherapy (CRT→S) vs. surgery alone

Before 2010, there were four main trials in this category where a majority of patients enrolled had AC histology compared to SCC. All of these trials used cisplatin and 5-flourouracil along with radiation therapy (RT) [12-14] except the trial by Urba *et al*. which used cisplatin, 5-fluorouracil and vinblastine along with RT [15]. The doses of RT ranged from 35 Gy [13] to 50.4 Gy [14]. In these trials, the majority of tumors were AC and located in the mid or distal thirds of the esophagus. Only two of the four trials showed a statistically significant survival advantage [12,14]; however, both of these trials are faced with criticism. The trial by Walsh *et al*. is widely criticized because of an unexpectedly low 3-year survival rate of 6% in the surgery alone arm versus 32% with trimodality therapy [12], compared to the 16-30% seen in the control arms of the other three trials, thus overestimating the clinical benefit of trimodality therapy. Both the trials of Walsh *et al.* and Tepper *et al*. were under accrued with small numbers of patients (total enrollment of 113 and 56, respectively) [12,14]. All four trials showed improvement in pathological complete response (pCR), but with wide variability, ranging from 12-40%. Given that these four trials were under-accrued, had conflicting outcomes, and were ‘contaminated’ with SCC tumors (despite having a majority AC), the relevance of the four randomized control trials for EGJ AC remained unclear and controversial. However, a metaanalysis that included these four trials along with 6 other neoadjuvant CRT trials, enrolling exclusively SCC patients, showed an overall benefit for all-cause mortality with HR 0.81 (95% CI 0.70-0.93, p = 0.002), and thus these data were used to support the use of neoadjuvant CRT for EGJ AC tumors [16]. An updated meta-analysis was recently reported, with HR 0.78 (95% CI 0.7-.088; p < 0.0001) [17].

However, most recently the large 363 patient CROSS trial was reported, which ultimately enrolled EC (SCC) and EGJ (AC) in a 1:3 ratio, evaluating CRT→S (carboplatin/paclitaxel/ 41.4Gy) versus S alone [18]. This was the first large adequately accrued ‘stand-alone’ phase III trial to support neoadjuvant CRT over surgery alone in this patient population. There was a pCR of 29% (49% for SCC, 23% for AC) and R0 resection rate of 92% versus 69% - for those patients actually receiving surgical resection (i.e. not intention-to-treat ITT). The overall mOS was 49.4 versus 24 months and HR was 0.67 (95% CI 0.45-0.87, p = 0.02). However, the subgroup analysis by histology was HR 0.422 (N = 88; 95% CI 0.24-0.79, p = 0.007) for SCC, and HR 0.741 (N = 275; 95% CI 0.53-1.02, p = 0.07) for AC. Despite these obvious clinically significant differences in outcome between histological subgroups, there was reportedly not a significant statistical interaction by histology. Based on these data, neoadjuvant CRT remains an accepted treatment option for EGJ AC tumors (Type I/II Siewert), particularly now with carboplatin/paclitaxel chemotherapy based on ‘CROSS’, and in particular in North America and the Netherlands. Awaited are the data detailing the differences in R0 resection rate, recurrence rates (local vs. distant), and 1, 3, 5 year survival rates between AC and SCC tumors between the two treatment groups. It is anticipated that the AC group will have derived less benefit from CRT, in terms of mOS, due to more distant recurrences as compared to SCC, despite improved local control.

### ii. Adjuvant chemoradiotherapy (S→C-CRT-C) vs. surgery alone

The landmark Macdonald/INT0116 trial randomized patients to receive adjuvant CRT (one cycle of bolus 5-FU monotherapy, followed by 2 cycles of 5-FU with concurrent RT (45Gy) for ~5 weeks, followed by 2 more cycles of 5-FU monotherapy) versus no adjuvant therapy in 556 patients with stage IB-IV (no distant metastasis, AJCC 6^th^ edition) GC (80%) or EGJ (20% type III Siewert) adenocarcinoma [19]. The regimen, referred to herein as the ‘MacDonald Regimen’, consisted of 5-FU and leucovorin administered before and after radiation. Chemotherapy (5-FU, bolus 425 mg/m2 per day, and leucovorin, 20 mg/m2 per day, for 5 days) was initiated on day 1 and was followed by CRT beginning 28 days after the start of the initial cycle of chemotherapy. CRT consisted of 45 Gy of radiation at 180 cGy per day, five days per week for five weeks, with bolus 5-FU (400 mg/m2 per day) and leucovorin (20 mg/m2 per day) on the first four and the last three days of radiotherapy. One month after the completion of radiotherapy, two five-day cycles of 5-FU (bolus 425 mg/m2 per day) plus leucovorin (20 mg/m2 per day) were given one month apart. The treatment completion rate was 64%. The trial showed improvement in mOS of 36 versus 27 months (HR 0.76, CI 0.62-0.92, p = 0.005), 3 year DFS (50 vs. 41%), and local recurrence rate (19 vs. 29%) with this adjuvant strategy. These data were recently updated and revealed a similar and persistent benefit, ensuring that adjuvant CRT with 5-FU remains a standard of care [20,21]. Of note, only 46% of the patients enrolled underwent ≥ D1 surgical resection (only 10% D2) [19]. A subset analysis showed benefit in all subgroups except diffuse-type histology [20,22]. Caveats of this trial include the small number (l0%) of patients undergoing D2 resection (and even D1 only 36%); it has been argued that the benefit gained by the addition of CRT merely offset the inadequate surgical approach (i.e. cleaning up a ‘bad surgery’). However, a retrospective analysis by Kim *et al.* compared 544 patients all receiving D2 resection followed by CRT for GC, to 446 receiving D2 surgery alone at their institution [23]. They reported that both mOS (95.3 vs. 62.6 months, p = 0.02) and mDFS (75.6 vs. 52.7 months, p = 0.0160) were significantly improved in the S→CRT group compared to surgery alone. A follow-up small phase III trial (N = 90) of stage III/IV (TNM-6) patients suggested that the addition of radiation therapy to chemotherapy could improve the locoregional recurrence-free survival (LRRFS) but not disease-free survival of gastric cancer treated with R0 gastrectomy and D2 lymph node dissection; a subgroup analysis of only stage III disease did significantly prolong the 5-year LRRFS and disease free survival rates compared with chemotherapy (93.2% vs 66.8%, p = 0.014; 73.5% vs 54.6%, p = 0.056, respectively) [24]. In contrast, another study reported no difference in survival in their single institution observational cohort (N = 142); a subgroup analysis of patients with LN + disease and higher N-ratio trended to improved clinical outcomes [25]. The negative ARTIST trial, discussed below, sought to prospectively evaluate the benefit of adjuvant CRT compared to adjuvant chemotherapy alone, in patients having a D2 lymphadenectomy, and also suggested benefit only within the LN + subgroup [26].

### iii. Neoadjuvant chemotherapy (C→S) vs. surgery alone

There are two main randomized controlled trials in this group (that include AC) [27,28], of which only one trial was statistically significant [27]. Both trials included SCC and both used preoperative chemotherapy with cisplatin (Cis) and 5-fluorouracil (5-FU) - but with different dosing regimens and schedules: 2 cycles Cis-80 mg/m2, 5FU 1000 mg/m2 days 1–4 administered every three weeks [27]; or Cis-50 mg/m2 D 1, 15, 29 and 5FU 2000 mg/m2 D1, 8, 15, 22, 29, 36 [28] - each followed by surgery and compared to surgery alone. The only positive trial was the OEO2 trial by the Medical Research Council (MRC) group, which randomized 871 patients (434 vs. 437), and enrolled ~64% of patients with EGJ AC (10% type III, 56% type I/II), the remainder being SCC of the esophagus. The HR for mOS was 0.78 (95% CI 0.67-0.93) and showed a statistically significant 2-year survival advantage with 43% versus 34% survival in favor of chemotherapy (P = 0.004) [27], without reported interaction by histology. The recent long-term update of OEO2 confirmed the survival benefit with preoperative chemotherapy with HR 0.84 (p = 0.03) and 5-year rate of 23% vs. 17.1% [29]. However, the EORTC 40954 trial, which planned to enroll 360 patients in order to detect a HR of 0.71 with 80% power and 2-sided significance level of 4%, ultimately closed early due to poor accrual with only 40% of the intended patients (72 per arm). As might be expected, the HR for mOS and mPFS were not statistically different at 0.84 (p = 0.466) and 0.76 (p = 0.2), respectively [28].

Differences in treatment dosing, ‘contamination’ with SCC histology, under accrual (EORTC 40954) and conflicting results of these two trials leave an approach of neoadjuvant 5FU/Cisplatin chemotherapy alone without definitive evidence to refute or support its use. The metaanalyses discussed above, which included both neoadjuvant CRT→S and C→S trials, revealed survival benefit to chemotherapy alone [16,17]. In the first meta-analysis, the hazard ratio for neoadjuvant chemotherapy was 0.90 (0.81–1.00; p = 0.05), with a 2-year absolute survival benefit of 7%. There was no significant effect on all-cause mortality of chemotherapy for patients with SCC (HR 0.88 [0.75–1.03]; p = 0.12), although there was a significant benefit for those with adenocarcinoma (0.78 [0.64–0.95]; p = 0.014) [16]. The updated meta-analysis showed a HR for all-cause mortality for all neoadjuvant chemotherapy patients (AC and SCC) of 0.87 [0.79-0.96; p = 0.005] [17]. The MRC OEO5 study is ongoing and evaluating two variables - a more aggressive triple drug regimen adding epirubicin (ECX) and for longer duration (4 cycles), versus CF (2 cycles) as in the OEO2 - results of which are awaited [30] (Table 2).

**Table 2:** Ongoing phase II and III trials in locally advanced gastroesophageal adenocarcinoma

**Trial**	**Setting**	**N (To be enrolled)**	**Location**	**Primary endpoint**	**Intervention**	**Expected completion date**
Alderson et al. [30], 2013	Neoadjuvant	842	Esophageal	Survival and QOL	A: C (ECX x 4) → S	NR
MRC OEO5			EGJ I-II		B: C (CFx2) → S	
Kang et al. [35], 2013	Adjuvant CRT	1000	Gastric and EGJ	DFS (3 years)	A: S → C (S1)	January 2016
NCT01761461					B: S → C (S1x1) → CRT (S1) → C (S1x6)	
‘ARTIST II’					C: S → C (SOX)	
					D: S → C (SOX) → CRT (S1) → C (SOXx4)	
Lorenzen et al. [53], 2010	Neoadjuvant CRT	NR	Resectable EGJ (I-II)		Non-randomized, single institution	Not initiated
NCT01271322					A: C (Cisplatin/Taxotere) → PET	
‘HICON’ Trial (Phase II)					If Response: Continue C	
					If No response: Cross to Arm B	
					B: Taxane based CRT (45 Gy) → PET	
					If Response: CRT (TP x 3)	
					If No response: Cross to Arm A	
Goodman et al. [54], 2013	Neoadjuvant CRT	204	Esophageal and EGJ I and II	pCR	A: C (FOLFOX x 3) → PET	September 2011
NCT01333033					If Response: CRT (FOLFOX x 3)	
CALGB 80803 (Phase II)					If No response: Cross to Arm B	
					B: C (TP x 3) → PET	
					If Response: CRT (TP x 3)	
					If No response: Cross to Arm A	
Nordwest et al. [74], 2012	Perioperative	590	Gastric EGJ I-III	DFS (2 years)	A: C (FLOTx4) → S (D2) → C (FLOTx4)	July 2015
NCT01216644					B: C (ECFx3) → S (D2) → C (ECFx3)	
‘FLOT4’						
Chen et al. [72], 2012	Perioperative	722	Gastric EGJ II or III	DFS (3 years)	A: C (SOX) → S (D2) → C (SOX)	September 2014
NCT01583361					B: S (D2) → C (SOX)	
‘RESONANCE’						
Shen et al. [73], 2012	Perioperative	1059	Gastric EGJ I-III	DFS (3 years)	A: S (D2) → C (SOXx8)	September 2014
NCT01534546					B: S (D2) → C (XELOXx8)	
					C: C (SOXx3) → S (D2) → C (SOXx5) → C (S-1x3)	
Reynolds et al. [60], 2013	Perioperative	366	Esophageal EGJ (I-III)	OS (3 years)	A: C (ECF) → S → C (ECF)	September 2021
NCT01726452					B: CRT (TP) → S	
‘MAGIC vs. CROSS EGJ’						
Verheji et al. [61], 2011	Perioperative CRT	788	Gastric	OS	A: C (ECCx3) → S (D1+) → CRT (CC, 45Gy)	June 2013
NCT00407186 CRITICS Trial					B: C (ECCx3) → S (D1+) → C (ECCx3)	
Leong et al. [62], 2013	Peri-operative CRT	752	Gastric EGJ	OS	A: C (ECF/Xx2) → CRT (5-FU/RT) → S → C (ECF/Xx3)	NR
‘TOP GEAR’ (Australia)					B: C (ECF/Xx3) → S → C (ECF/Xx3)	
**Molecularly targeted trials**						
Safran et al. [67], 2013	Neoadjuvant CRT	480	Mid and Distal Esophagus including EGJ	DFS	A: CRT (TP) → S → T (13)	August 2018
NCT01196390					B: CRT (TP) → S	
RTOG 1010						
Reed et al. [68], 2012	Neoadjuvant CRT	69	Distal Esophagus and EGJ	pCR	CRT (CDP) → S	Study Completed
NCT00757172						
ACASOG Z4051						
Phase II						
Ilson et al. [69], 2012	Neoadjuvant CRT	420	Esophageal (Squamous allowed) GEJ I-II	OS	A: CRT (CCT, 50.4 Gy)	August 2018
NCT00655876					B: CRT (CT, 50.4 Gy)	-Closed at interim analysis for AC arm due to low clinical complete response
RTOG 0436						-Closed SCC arm due to SCOPE-1 results (see text)
Cunningham et al. [75], 2012	Perioperative	1100	Gastric EGJ I-III	OS, Safety, Efficacy	A: C (ECXB) → S → C (ECXB)	December 2014
NCT00450203						
‘MAGIC-B’ (ST03)					B: C (ECX) → S → C (ECX)	

*Abbreviations:PET* positron emission tomography; *FLOT* 5-flourouracil, leucovorin, oxaliplatin and docetaxel; *FOLFOX* 5-FU, leucovorin and oxaliplatin; *XELOX* oxaliplatin with capecitabine; *ECC* epirubicin, cisplatin, capecitabine; *CDP* cisplatin, docetaxel, panitumumab; *CCT* cetuximab, cisplatin, and paclitaxel; *SOX* S-1 and oxaliplatin; *TP* paclitaxel and carboplatin; *T* trastuzumab; *B* bevacizumab; *QOL* Quality of Life; *5-FU/L* 5-flouroacil and leucovorin; *RT* radiation therapy; *XP/RT* capecitabine/cisplatin/radiotherapy; *NR* not reported.

### iv. Adjuvant chemotherapy (S→C) vs. surgery alone

In the ACTS-GS trial from Japan, 1059 GC patients (50% Stage II, 40% Stage III AJCC 6^th^ edition), who all underwent D2 dissection, were randomized to either observation or S-1 chemotherapy (40 mg/m2 BID x 4 weeks, 2 weeks off) for 1 year [31,32]. Of note, 97% of patients had a T2 or T3 lesion and 90% of patients had N1 or N2 disease. There was a significant improvement in mOS with S-1 chemotherapy compared to observation that was maintained in the 5-year update with HR 0.67 (95% CI 0.54-0.828) and 5-yr DFS 71.7% vs 61.1%, respectively. The authors concluded that S-1 is an effective adjuvant treatment after D2 dissection in East Asian patients with locally advanced GC [31,32].

Paoletti *et al.* conducted a large metaanalysis of 17 RCTs with individual patient data for 3838 patients to evaluate the efficacy of adjuvant chemotherapy (heterogeneous regimens) compared to surgery alone. They reported an absolute 5- year overall survival advantage of 5.7% with adjuvant chemotherapy compared to surgery alone (55.3 vs. 49.6% respectively, HR 0.82, CI 0.76-0.90, P = 0.001) with similar HR for DFS [33].

Recently, the ‘CLASSIC’ trial was reported by Bang *et al.* in stage II-IIIB GC patients [34]. All patients underwent curative D2 gastrectomy and were enrolled from 37 centres in South Korea, China, and Taiwan. Patients were randomized to either observation or adjuvant chemotherapy with 8 cycles of CapeOx (oral capecitabine, 1000 mg/m2 BID on D 1–14 of each 3 week cycle, plus oxaliplatin 130 mg/m2 on D1 of each 3 week cycle) for 6 months. The trial showed significant improvement in the 3-yr OS (83% vs. 78%, HR 0.72, 95% CI 0.62-1; p = 0.0493) and 3-yr DFS with adjuvant CapeOx compared to surgery alone (74 vs. 59%, HR 0.56, 95% CI 0.44-0.72; p < 0 · 0001); mature data for robust estimates of mOS are awaited. The adjuvant chemotherapy was associated with more grade ≥ 3 toxicity compared to surgery alone (56% vs. 6% respectively) with nausea, neutropenia, and decreased appetite as the major toxicities [34]. The four arm ‘ARTIST-II’ trial, assesses S1 monotherapy versus S1 and oxaliplatin (SOX) with or without CRT (with concurrent S1) in LN + disease only [35] (Table 2).

### v. Perioperative ‘sandwich’ chemotherapy (C→S→C) vs. surgery alone

The trial, INT-0113/RTOG8911, by Kelsen *et al.* randomized 467 patients (53% AC) to surgery alone or to 3 monthly preoperative cycles and 2 post-operative cycles of Cisplatin 100 mg/m2 and 5FU 1000 mg/m2 days 1–5 [36,37]. This resulted in no difference in survival (p = 0.53) [36], yet updated results revealed that patients who responded to chemotherapy radiographically (n = 39, 19% total, 12% PR, 7% CR) had a substantial improvement in long term survival, while the non-responders did not differ significantly from those undergoing surgery alone, a phenomenon that was similar to the MUNICON studies discussed below. Moreover, R0 resection proved to be a major determinant of long-term survival, and the chemotherapy (ITT) group had a R0 rate of 63% versus 59% (ITT) [36,37]. Of note, in multivariate analysis, for patients assigned to chemotherapy and surgery, not responding to chemotherapy (HR, 2.83; 95% CI, 1.84 to 4.35; p<0.0001) and >10% weight loss (HR, 1.47; 95% CI, 1.09 to 1.98; p *=*0 .0109) were associated with increased risk of death, while adenocarcinoma histology (HR, 0.59; 95% CI, 0.44 to 0.80) was associated with decreased risk of death. Only 70% of patients received all three cycles of preoperative therapy, and only 30% of patients received at least one post-operative cycle [36,37].

In contrast, two large randomized controlled trials, ‘MAGIC’ and the FNLCC ACCORD07-FFCD9703 trial, established the role of perioperative chemotherapy in improving survival compared to surgery alone [38,39]. Both of these trials included only patients with AC histology, with ~25% and 75% EGJ tumors in ‘MAGIC’ and FFCD trials, respectively.

The ‘MAGIC’ trial enrolled 503 patients randomizing to 3 cycles of ECF before and 3 cycles after surgery, compared to surgery alone. This resulted in an improvement in mOS with HR 0.75 (95% CI 0.60-0.93, p = 0.009) and 5-yr survival rate of 36% versus 23% compared to surgery alone. There was a similar benefit in PFS with HR 0.66 (95% CI 0.53-0.81, p < 0.001). Only 66% of the patients started post-operative chemotherapy and 76% of these patients actually completed the planned course of chemotherapy; only 41.6% of patients completed all 6 cycles of therapy. This is a significantly higher completion rate than in the INT-0113 trial, discussed above [36,37]. There was a significant trend to having fewer patients in the advanced T-stage (p = 0.002) and N + groups (p = 0.01) within the perioperative chemotherapy group, despite randomization. Only 41% of patients received a D2 resection, (D1 19%, D0 40%). R0 resection was observed in 79% versus 69% of patients in favor of the chemotherapy arm (ie. not ITT). It should be noted that 91.6% of patients in the C→S→C arm underwent surgery, while 96.4% underwent surgery in the control arm. More patients did not proceed to surgery in the chemotherapy arm (6.1%) versus the surgery alone arm (2.4%), many of which progressed during the neoadjuvant chemotherapy phase, thus excluding them from curative-intent surgery. It can be argued that this neoadjuvant approach delaying immediate resection actually spared this subset of patients with aggressive disease from unnecessary surgery, which would have unlikely changed the ultimate outcome.

The ACCORD07 trial randomized patients to cisplatin (100 mg/m2) and 5FU (800 mg/m2 D1-5) every 28 days (N = 113) for up to 3 cycles prior to surgery and up to 4 cycles after surgery, versus surgery alone (N = 111) (D2 recommended) [39]. The trial slightly underaccrued from its target enrollment of 250 patients. Most patients in the chemotherapy arm received 2 cycles preoperatively (78%), and 87% of patients received at least 2 preoperative cycles. Among those who received at least 1 cycle of preoperative chemotherapy (n = 109), 54 patients (50%) received at least 1 cycle of post-operative therapy (6/7/16/25 patients received 1/2/3/4 cycles postoperatively, respectively). Of those who relapsed (55% vs. 64%), locoregional only relapse was low (12 vs. 8% in the chemotherapy vs. surgery alone, respectively), compared to the aggregate of distant relapse and both distant/local relapse (42% for chemotherapy, 56% for surgery alone). This emphasized the systemic nature of GEC. The mOS was improved with HR 0.69 (95% CI 0.50-0.95, p = 0.02) with 5-yr survival rates of 38% vs. 24%. DFS similarly was improved with HR 0.65 (95% CI 0.48-0.89, p = 0.003) with 5-yr DFS rates of 34% vs. 19%. R0 resection rate was observed in 84% vs. 74% of patients. The subgroup analysis of EGJ patients (comprising 75% of accrued patients) for mOS, revealed a HR 0.57 (95% CI 0.39-0.83). Caveats to both the ‘MAGIC’ and ‘ACCORD-07’ trials were lack of pre-treatement staging as endoscopic ultrasound was as yet unavailable. Given very similar results between ‘MAGIC’ and ‘ACCORD-07’, the additional benefit of epirubicin in this setting has been questioned, which may be potentially addressed in the ongoing OEO5 trial (without a post-operative chemotherapy component), discussed above.

Interestingly, phase II reports with FLOT and DCF ‘sandwich’ chemotherapy have reported pCR rates of 10-12%, and R0 rates ranging ~85-100%, supporting the ongoing evaluation of these regimens in larger trials [40-42] (Table 2).

With similar survival benefits as measured by HR and absolute 5-year survival rates of the ‘sandwich’ approach to neoadjuvant CRT for EGJ AC, perioperative treatment with ECF or CF are accepted standards, particularly in Britain, Europe and some centers in North America.

### vi. Comparison of post-operative chemotherapy regimens

#### S→FOLFIRI then DC vs S→5FU

The ITACA-S trial evaluated whether intensification of postoperative chemotherapy would improve outcomes of patients undergoing curative intent resection of GC or EGJ AC. Patients with ≥ D1-lymphadenectomy were randomized to irinotecan 180 mg/m^2^ on d1, LV 100 mg/m2 d1-2, 5-FU 400–600 mg/m2 d1-2, q14; (FOLFIRI regimen) for 4 cycles, followed by docetaxel 75 mg/m2 d1, cisplatin 75 mg/m2 d1, q 21; (DC regimen) for 3 cycles (arm A) as compared to the ‘control’ arm of LV 100 mg/m2 d1-2, 5-FU 400–600 mg/m2 d1-2, q 14 for 9 cycles (arm B). Patients were randomized (562 arm A, 538 arm B) by 123 Italian centers. The primary endpoint of DFS was not statistically different between the groups (HR 0.98; 95% CI 0.83-1.16; p = 0.83), nor was there an observed difference in mOS (HR 1.00; 95% CI 0.83-1.20; p = 0.98). The authors concluded that adjuvant chemotherapy for GC/EGJ with a more intensive regimen did not result in a significant prolongation of DFS and OS when compared to the bolus/infusion FU/LV regimen, suggesting that intensification of chemotherapy may not improve outcomes [43].

#### (S→UFT vs S→S1 vs S→paclitaxel then UFT vs. S→ paclitaxel then S1)

The four arm SAMIT trial was a phase III trial with a two-by-two factorial design planned to assess the survival benefit of adjuvant chemotherapy intensificaiton via sequential use of paclitaxel and oral flurinated pyrimides (FP) in comparison to FP alone, and to compare the FPs tegafur/uracil (UFT) versus S-1. An initial report showed that adjuvant chemotherapy with various combinations of taxol and fluoropyrimindines was well tolerated [44]. Recently, the final results were reported, where the primary endpoint of DFS revealed that UFT was inferior to S-1 (HR 1.23, 1.07-1.43), and that there was a nonsignificant trend (HR 0.92, 0.8-1.07; p = 0.23) to improved DFS with sequential PTX→FP versus FP alone [45].

These two trials, SAMIT and ITACA-S, suggest that intensification of chemotherapy does not improve survival over fluoropyrimidine alone.

### vii. Adjuvant CRT comparing peri-RT chemotherapy regimens

#### (S→5FU-CRT-5FU vs S→ ECF-CRT-ECF)

The CALGB 80101 trial by Fuchs *et al*. compared post-operative CRT with intensified peri-RT chemotherapy using ECF versus the standard 5FU treatment [46]. However, the control arm received a modified ‘Macdonald regimen’, with continuous infusion (CI) 5FU 200 mg/m2 everyday through to completion of radiation (45Gy), rather than the original bolus 5FU concurrently with RT. The investigational arm received 1 cycle of ECF chemotherapy before and 2 cycles ECF after CRT, in 546 patients with GC or EGJ AC (~33%). The trial did not show any significant difference between the ECF and 5-FU arms for mOS (HR, 1.03; 95% CI, 0.80-1.34; p = 0.8) or 3-yr DFS (52 vs. 50% respectively). Counter-intuitively, the toxicity comparison between the 5-FU arm compared to the ECF arm revealed a slightly higher treatment related mortality (3% vs. <1% respectively) as well as grade 4 toxicity (40% vs. 26% respectively, p < 0.001); this may be attributed to the different peri-RT chemotherapy dose schedules of the 5FU between the arms (bolus ‘Macdonald regimen’ versus continuous infusion). The authors concluded that there is no survival advantage of postoperative CRT using intensified peri-RT ECF compared to 5-FU [46]. The authors also indicated that there was an approximate 40% D2 resection rate, but that accurate D0/D1/D2 resection rates will be reported at a later date [46].

### viii. PET directed therapy

Sarkaria *et al.* conducted a retrospective study using a prospectively maintained database to determine if endoscopic biopsy after neoadjuvant CRT therapy would predict pathological CR (pCR) [47]. It was observed that a negative endoscopic biopsy was not reliable for predicting pCR, nodal stage or overall survival. Overall, PET imaging for predicting pathologic response in EGJ has been most promising. In a retrospective study, Weber and colleagues first established that a SUV decrease of ≥35% from baseline was predictive of 2-year survival with a sensitivity of 93% and specificity of 95% [48]. The SUV cut-off was subsequently validated prospectively by Ott *et al.*[49]. They also found that metabolic responders showed a high histopathologic response rate of 44% compared to only 5% in metabolic non-responders (p = 0.001) [49]. This led to a single center, exploratory, phase II ‘MUNICON’ study in patients with EGJ cancer [50]. Enrolled patients (N = 119 EGJ I/II) were originally staged as cT3 or cT4 based on CT, EUS, and PET. A repeat PET scan at day day 14 into treatment, as compared to baseline, was done to assess the metabolic response to chemotherapy (cisplatin and 5-FU). Responders continued with chemotherapy for 12 weeks, while non-responders proceeded directly to surgery. In metabolic non-responders, mOS was 25.8 months (19.4–32.2), whereas mOS was not reached in responders after a follow-up of 2.3 years (HR 2.13, CI 1.14–3.99, p = 0.015). A major histological remission (<10% residual tumor) was found in 58% of metabolic responders, compared to none in metabolic non-responders. R0 resection rates were 82% in PET-responders, and 70% in non-responders. Importantly, a comparison of these observed results to historic cohorts showed that discontinuing inactive chemotherapy after 2 weeks, and avoiding delay in surgery, did not adversely affect outcomes [51]. Based on these results, the ‘EUROCON’ trial planned to randomize metabolic nonresponders after 2 weeks of chemotherapy to immediate resection or CRT followed by surgery, but this trial did not materialize. However, the smaller ‘MUNICON-II’ trial was completed (N = 56, EGJ I/II) where PET-responders were continued on chemotherapy alone (cisplatin 50 mg/m2 days 1, 15, and 29; 5FU 2000 mg/m2 over 24 hours days 1, 8, 15, 22, 29, repeated on day 49; folinic acid 500 mg/m2 over 2 hours with each 5FU administration), while non-responders proceeded with neoadjuvant CRT prior to surgery [52]. This design had the power to detect an improvement in the R0 resection rate from 74% to 94% in non-responders (1-sided alpha 0.1, power 0.8), with requirement of treating 23 non-responders with salvage neoadjuvant CRT (32 Gy with 1.6 Gy twice daily and 10 fractions per week; cisplatin 6 mg/m2 days 1–5 and 8–12 OR 5FU 250 mg/m2 CI if renal function inadequate). Of the 56 patients enrolled, 23 were non-responders and obtained ‘salvage’ CRT prior to surgery. The 2-year survival rates were 74% vs 57% (p = 0.035) in favor of the PET-responders. Although there was an increased histopathological response observed in the ‘MUNICON-II’ salvage CRT non-responder group (as compared to the ‘MUNICON I’ trial histopathological rate, where non-responders proceeded directly to surgery without further neoadjuvant treatment), the primary endpoint was not reached in ‘MUNICON-II’ with an observed R0 resection rate of 74%, not significantly better than the R0 rate of 70% observed in ‘MUNICON-I’. ‘MUNICON-II’ confirmed the poor prognosis of PET non-responders (which is approximately 50-60% of EGJ I/II patients), despite aggressive salvage neoadjuvant CRT. The authors concluded that despite salvage neoadjuvant CRT in PET-nonresponders leading to local remissions in 6 (16%) of patients, it was not able to change the clinical course in general because of the systemic disease recurrence patterns. They also surmised that improvement of distant recurrence would be required to improve outcomes for this patient subset. Criticism of the MUNICON-II trial is the relatively low dose of radiation, as well as the single agent Cisplatin (or 5FU) during CRT, which is less aggressive than desired for optimal local control.

In addition to the ‘MUNICON-I and -II” trials, the ‘HICON’ and CALGB 80803 clinical trials plan to evaluate PET-directed treatment in the neoadjuvant setting prior to surgery for EGJ, as discussed below [53-55]. Unfortunately, the HICON trial was never initiated. Moreover, PET directed therapy is being evaluated for locally advanced GC where patients with baseline PET tumor SUV > 3.5 (or tumor : liver ratio > 1.5) receive one cycle (C1) epirubicin (50 mg/m2), cisplatin (60 mg/m2) day and capecitabine (625 mg/m2 twice daily days 1–21) (ECX) with bevacizumab (15 mg/kg day 1) therapy preoperatively for 21 days, with a repeat post-C1 PET. Patients with a metabolic response (>35% decrease in FDG uptake) on repeat PET proceed with two further cycles of the initial treatment. PET nonresponders switch to ‘salvage’ docetaxel (30 mg/m2) and irinotecan (50 mg/m2) Days 1 and 8 every 3 weeks, along with bevacizumab (15 mg/kg day 1), for two cycles. Postoperatively, patients continue on the chemotherapy regimen they received just prior to surgery for three more cycles [56]. Twenty of 60 planned patients were enrolled before closure of this trial to poor accrual; 11/20 had PET response. Ten of 11 (91%) responders achieved R0 resection and 4/11 (36%) achieved pathological responses (1 pCR, 3 pPR). Seven of 9 (77%) PET non-responders achieved R0 resection; none achieved a pathological response. There was no significant difference in DFS between the responders and non-responders (p = 0.4). This hypothesis generating pilot trial demonstrated that PET imaging during induction chemotherapy can identify early treatment failures, with potential benefit of altering to salvage chemotherapy. This has led to the proposal of a larger Cooperative Group trial currently under consideration.

### ix. Induction chemotherapy followed by chemoradiation

#### (C→CRT→S vs. C→S; C→CRT→S vs. CRT→S)

One reported trial, ‘POET’, assessed the benefit of induction chemotherapy followed by concomitant CRT in randomized fashion versus chemotherapy alone, (unlike ‘MUNICON-II’, discussed above, where only PET-non-responders of ‘induction’ chemotherapy proceeded with CRT) [50]. The ‘POET’ trial, used induction chemotherapy with PLF (cisplatin, leucovorin and 5-fluorouracil), comprised of cisplatin 50 mg/m2 biweekly and weekly 5FU 2000 mg/m2 with leucovorin (one course 6 weeks) [57]. The investigational arm completed 2 courses of PLF followed by RT with concurrent PE (cisplatin 50 mg/m2 D 1,8 and etoposide 80 mg/m2/day D 3–5) compared to the ‘control arm’ of chemotherapy alone using PLF for 2.5 courses (15 weeks) followed by surgery. This trial, then, was comparing neoadjuvant chemotherapy with/without CRT prior to surgery. Unique inclusion criteria included only high-risk T3-4 patients with EGJ AC (Siewert’s I-III). The trial required pre-operative staging with EUS and laparoscopy. Unfortunately, the accrual goal was not met (only 33% of the planned patients were recruited, N = 119). The trial showed a trend towards improvement in 3 year OS with induction chemotherapy followed by CRT (47 vs. 28%, respectively) but it was not statistically significant (HR 0.67, CI 0.41-1.07, p = 0.07). Additionally, the pCR rates (16 vs. 2%) and local control (77 vs. 59%) were better in the investigational CRT arm compared to the chemotherapy alone control [57]. The comparison of induction chemotherapy prior to CRT versus chemotherapy alone seemed promising in this trial, albeit using an unconventional chemotherapy of etoposide during RT. However, from the ‘MUNICON-II’ trial (with recognized caveats of potentially underdosed RT and inadequate concurrent chemotherapy with cisplatin alone), it appeared that non-responders to chemotherapy alone did not derive large therapeutic benefit from further neoadjuvant CRT in terms of survival or R0 resection rates, while chemotherapy responders had improved outcomes. Therefore, it is possible that chemotherapy responders may be treated optimally with chemotherapy alone, without deriving benefit from additional CRT. To prospectively study this, a trial exclusively evaluating chemotherapy responders randomized to continued chemotherapy versus intensified CRT could be done, in order to determine if CRT adds anything to those already responding to chemotherapy alone. Similarly, to evaluate the utility of induction chemotherapy prior to CRT (assuming the need for CRT), a prospective randomized trial of induction chemotherapy prior to CRT versus CRT alone would be required. One such trial, N0849, was recently reported, with the primary endpoint assessing pCR rate [58]. The interim report on efficacy and futility revealed a pCR rate of 7/21 (33%) in the induction chemotherapy arm versus 10/21 (48%) in the CRT alone arm, and R0 resection rates of 16/17 (94%) versus 20/20 (100%), respectively. The authors concluded the induction chemotherapy failed to improve pCR rates, yet followup in regard to survival and rate (and site) of recurrence is ongoing [58]. One might suggest that addition of induction chemotherapy would not be expected to improve pCR rates, but rather and if anything, decrease distant recurrence rates - data which are currently not mature. Regardless, the principle of C→CRT prior to surgery for all patients, whether there is response or not to the induction chemotherapy, is being evaluated in CALGB 80803 trial using a PET directed algorithm with chemotherapy backbone cross-over if there is demonstrated lack of PET response - intentions of optimizing both local (RT) and distant (chemotherapy) control [54] (Table 2).

### x. Adjuvant chemotherapy versus CRT (S→C vs. S→C-CRT-C)

In addition to the small trial by Kim *et al.* above [24], Lee *et al*. treated 458 resected GC patients with post-operative chemotherapy in the ‘ARTIST’ trial, with 2 cycles of XP (capecitabine and cisplatin), followed by concurrent capecitabine with RT, followed by 2 more cycles of XP, compared to post-operative chemotherapy consisting of 6 cycles of XP alone [26]. Again, all patients underwent D2 gastrectomy and had R0 resection of GC (only 5% type III EGJ). The primary endpoint of the trial, DFS, was not met with 3-yr DFS rates available at the time of reporting (78.2% vs. 74.2%; P = 0.0862). However, a subgroup analysis of LN + disease (N = 396, 86%) did reveal improved DFS with HR 0.6865 (95% CI 0.47-0.99; P = 0.047) [26]. The authors, due to the nature of subgroup analyses, caution regarding conclusions with respect to LN + disease, and indicate that a subsequent trial, ‘ARTIST-II’, will prospectively evaluate the utility of C→CRT→C versus chemotherapy alone (S1 versus S1 and oxaliplatin - SOX) in LN + disease undergoing D2 resection in a four-arm trial [35]. The ‘ARTIST-II’ trial will evaluate the question of intensification in LN + only disease. The results from ‘ARTIST’ suggest that low-stage (N-) disease may be adequately treated with modified D2 resection without the need for CRT.

## The devil is in the details: which perioperative approach?

Adding the recent trials ‘CROSS’, CALGB 80101, ‘CLASSIC’, ‘ITACA-S’, ACCORD07 and ‘ARTIST’ to the repertoire of pre-2010 studies evaluating various perioperative treatment strategies, no regimen has emerged with international consensus for GC or EGJ AC cancers. Excluding the ‘ITACA-S’, SAMIT, ‘ARTIST’, CALGB80101, and ‘POET’ trials, all of the discussed relevant stand-alone phase III trials compare the investigational arm to surgery alone, making direct comparison of differing perioperative approaches complicated. We are limited to cross-trial comparisons and meta-analyses, and the caveats and shortcomings that this entails, including differences in enrollment criteria and actual enrollment variances, such as tumor location (i.e. GC/EGJ mix) (Figure 1). For instance, EGJ AC tumors are less-well represented within S→CRT and S→C trials, whereas they do comprise a more substantial subset within C→S→C, C→CRT→S, and CRT→S trials. Additionally, differences in histology (ie AC +/− SCC), stage (eg. most enrolled ≥ stage II, but some enrolled ≥ stage Ib; whether EUS and/or staging laparoscopy/washings were performed), and other nuances such as proportion of D1/D2 surgery, chemotherapy and radiotherapy type, dose and/or schedule, and geographic location/ethnic makeup variances from trial to trial, all make cross-trial comparisons quite problematic.

While there is consensus that ‘something-other-than-surgery-alone’ should be done for GEC, and in particular EGJ AC, with consistent clinical improvement observed regardless of the perioperative approach chosen (~10-15% absolute 5-yr survival benefit, HR 0.60-0.80), opinions regarding C→S→C vs CRT→S differ, and are formulated based on cross-trial comparisons and clinician experiences. For patients seeking second/third opinions, this can be particularly confusing when they receive different opinions regarding treatment strategy. The ‘CROSS’ authors favor CRT→S for EGJ AC, stating that the ‘ACCORD-07’ and ‘MAGIC’ trials have a heterogeneous distribution of GC and EGJ patients, as well as citing the under-accrued relatively small ‘POET’ trial as supporting arguments [18]. However, the ‘POET’ trial was not a comparison of C→S→C to CRT→S, but rather C→S to C→CRT→S, and had unique T-stage eligibility (Table 1), used non-standard CRT with etoposide, under-accrued, and ultimately, had a non-significant survival difference. Moreover, ‘ACCORD-07’ accrued 75% EGJ patients, and subset survival analysis of these EGJ patients showed a HR 0.57 (0.39 to 0.83), albeit without statistically significant interaction by tumor location, possibly due to lack of power of this analysis. Furthermore, the ‘CROSS’ trial also had significant differences in eligibility, namely, histology (SCC 25%); despite the statement that benefit was consistent across histologic subgroups without interaction, the AC patients clearly demonstrated less benefit in terms of pCR, recurrence, and survival (HR 0.74 (0.53-1.02)). Finally, as demonstrated by the relapse patterns of the ‘ACCORD07’ and ‘MUNICON’ trials, distant recurrence is the apparent challenge for EGJ AC, for all patients, whether treated with chemotherapy, CRT or surgery alone, underscoring the systemic nature of EGJ AC. Until superior approaches are available that diminish distant recurrence, local control will be less important in terms of survival benefit, in contrast to the SCC histology. An important exception includes the scenario when R0 resection is deemed unattainable without RT prior to surgery. Again, although the pCR rate with neoadjuvant chemotherapy alone is low, ranging from 2-7% [36,38,39,57], these lower pCR rates, as compared to CRT (20-25%), appear to be less important than R0 resections in predicting long-term survival. With chemotherapy alone, absolute R0 rates are improved over surgery alone approximately 10% (eg. ACCORD 07: 74% to 84%, MAGIC 69% to 79%). Despite these rates being lower than those of CRT→S in some trials (CROSS 68 to 90% for AC) compared to surgery alone, the HR and absolute 5-year survival rates are similar between the two strategies. In contrast, R0 resections rates were not significantly different in the POET trial comparing C→S to C→CRT→S (70 vs 72%, respectively; Table 1) [57]. However, if RT is determined to be required to optimize R0 resection at multi-disciplinary tumor board, such as with T4 tumors, then it is appropriate to proceed accordingly [59]. Therefore, in general, higher pCR and even R0 resections seen with neoadjuvant CRT do not correlate with long-term overall survival improvement in comparison to ‘sandwich’ chemotherapy strategies (with the limitations of cross-trial comparisons). This is likely a function of the systemic propensity of GEC and ultimate distant recurrence. Moreover, as discussed above in PET-directed therapy, the MUNICON II trial may provide an explanation for the apparent lack of significant survival differences between the CRT→S and C→S→C strategies. It is plausible that any of the survival benefits observed in the various CRT→S trials could be attained with chemotherapy alone (either C→S or C→S→C). This is akin to the chemotherapy-responders in MUNICON II. On the other hand, for chemotherapy-nonresponders, it doesn’t matter what one does, the survival outcome is unchanged (hence no need for RT at all, in either scenario). Prospective evaluation of these questions are warranted to assess these pertinent questions. Finally, more aggressive chemotherapy with FLOT and DCF ‘sandwich’ regimens have reported higher pCR rates of 10-12% and R0 rates of ranging 85-100% than previous ECF or CF regimens, which merit further testing in several ongoing trials (Table 2) [40-42].

Cross-trial comparisons to determine best treatment approach must be done understanding key differences in eligibility criteria of the trials in question – particularly primary tumor location (Figure 1), stage, and histology – as well as surgical technique, pre-treatment staging and nuances of chemoradiotherapy type, dose, and schedule. The Devil is in the details.

Evidence that there is lack of consensus for optimal perioperative treatment of GEC, and in particular EGJ, is reflected by a number of current ongoing trials (Table 2). Fortunately, some of these trials are directly comparing the various perioperative strategies head-to-head. These include ‘RESONANCE’ and ‘SOX’, trials which are evaluating the ‘sandwich’ chemotherapy approach (with various chemotherapy backbones) compared to post-operative chemotherapy alone. The ‘ARTIST II’, as discussed above, seeks to confirm, prospectively, the perceived post-operative CRT benefit within the ‘ARTIST’ LN + subgroup in patients undergoing R0 D2 resection, as well as the intensification of chemotherapy question (SOX vs S-1) in this population in a 4-arm trial [35].

Currently, meta-analyses, such as Sjoquist et al., fail to definitively demonstrate superiority of CRT to chemotherapy alone, with the acknowledged numerous caveats of such comparisons (HR 0.88 (0.76-1.01; p = 0.07)) [17]. Supporters of CRT point to trends in survival benefit, whereas supporters of chemotherapy alone argue that there are no definitive data to support significant superiority (statistically or clinically) of CRT over chemotherapy alone. To answer the question of whether C→S→C or CRT→S is best for EGJ, the planned 366 patient ‘MAGIC vs. CROSS Upper GI’ trial randomizes patients with AC of the EGJ (I/II/III) to the standard ‘MAGIC’ or ‘CROSS’ regimen [60]. Finally, the addition of RT to the perioperative ‘sandwich’ approach is being evaluated in direct comparison to the standard ‘sandwich’ approach in the ‘CRITICS’ (RT post-operative, >D1 surgery, GC and Type III EGJ, N = 788) [61] and ‘TOP GEAR’ (RT pre-operative, D2 or D1+ for EGJ requiring an esophago-gastrectomy, GC and Type II/III EGJ excluding tumors that involve >2 cm distal esophagus, N = 752) [62]. Results of each of these pivotal large randomized phase III trials will be eagerly awaited and will optimistically clarify a number of uncertainties faced currently in this arena. Of course, it is likely that new questions will arise from these studies, and very possible that results may not establish a consensus approach for the perioperative treatment of GEC, if outcomes are similar between comparator groups. Should this be the case (that CRT versus chemotherapy are not substantially different in outcome), other factors such as patient quality of life (ie. requiring daily treatment for ~5 weeks versus chemotherapy every 2–3 weeks, toxicity differences, etc.) and/or treatment regimen cost will likely play a role in establishing treatment standards.

## Addition of molecularly targeted agents to perioperative therapy

The integration of ‘molecularly targeted’ agents to perioperative GEC therapy is complicated, again, by the various available ‘backbone’ strategies reviewed above. Addition of novel agents to neoadjuvant CRT are underway with anti-EGFR (cetuximab, SCOPE 1 and RTOG0436; panitumumab, ACASOGZ4051), anti-HER2 for HER2+ tumors (trastuzumab, RTOG1010), and anti-angiogenesis (bevacizumab) [63-69]. The RTOG0436 trial, evaluating ~350 patients with EGJ and EC (AC and SCC) with paclitaxel, cisplatin, RT with/without cetuximab, revealed only a modest endoscopic CR rate which triggered an early stopping rule in AC histology; the trial was then open for SCC only [68]. However, a recently reported study observed treatment-related fatalities and inferiority to standard treatment with the addition of cetuximab to CRT in the SCOPE 1 (C→CRT +/− cetuximab →S; 73% SCC esophagus, 27% EGJ AC) trial [63], and served as the basis to fully close all arms of RTOG0436. This observation of potential intolerable toxicity and/or survival detriment with the addition of novel molecularly targeted agents to RT in this setting may hinder further development along these lines [70,71].

On the other hand, introducing novel agents to chemotherapy alone may have less toxicity, and therefore it may be more conducive to evaluate these agents along with perioperative approaches that do not incorporate RT, particularly given the above arguments that there has not been definitive evidence to suggest superiority of CRT based regimens versus non-CRT regimens. A number of studies (MRC OEO5, NCT01761461, NCT01534546, NCT01583361, and FLOT4) [30,35,72-74] are underway (Table 2), evaluating for optimal ‘backbone’ chemotherapy regimens and sequence. Trials, including ST03, (aka ‘MAGIC 2’, ECX+/−bevacizumab) [75], seek to address the question of benefit of targeted agents added to chemotherapy backbones. There is also continued evaluation of the PET directed strategies discussed above [49-53,55].

Due to several negative trials in the metastatic setting, the SCOPE-1 results, and the lack of significant clinical CR rate for AC observed in RTOG0436, further evaluation of anti-EGFR therapy is unlikely to be pursued in the perioperative setting, at least in unselected patients. Additionally, there is suggestion that there may be a negative interaction, or at best no improvement, of adjuvant C→CRT→C in HER2+ patients within the INT-0116 trial, albeit with very small numbers [76]. Along with RTOG 1010 evaluating trastuzumab in neoadjuvant CRT approach, it is anticipated that anti-HER2 therapies will be evaluated in HER2+ EGJ/GC patients using the ‘sandwich’ chemotherapy backbone. However, difficulties in accrual to perioperative clinical trials are exacerbated by the low frequencies of molecular ‘oncogenic drivers’; this is the case for HER2+ disease, comprising only 10-20% of GEC. Potentially ‘actionable’ oncogenic drivers including MET, KRAS, PIK3CA, FGFR2, SRC and others have even less frequent genomic activation in GEC (Table 3). Therefore, there is great need for novel clinical trial designs and strategies using medium throughput technologies and treatment algorithms with access to multiple therapeutic agents within the same trial, in order to address this increasingly recognized challenge [77].

**Table 3:** **Medium-throughput targeted sequencing**^**Т**^**of GEC patients undergoing curative intent resection demonstrating profound interpatient molecular heterogeneity**

**Patient**	**Oncogene**	**Tumor suppressor**
**1**	HER2 Amp+, SRC Amp+, TOP1 Amp+	
**2**	FGFR2 Amp+	TP53 mt, CDH1 mt
**3**	SRC Amp+, AURKA Amp+, CCND1 Amp+, CDK4 Amp+, RICTOR Amp+, MDM2 Amp+	CDKN2A/B Loss, ATM mt
**4**	HER2 Amp+, PIK3CA mt, CDK6 Amp+	TP53 mt, PTEN mt
**5**	MDM2 Amp+	
**6**	MYCN Amp+	TP53 mt, FANCA Loss
**7**	PIK3CA mt	
**8**		ARID1A mt, ARID2 mt, Smad2 mt, MLL2 mt
**9**	HER2 Amp+, PIK3CA mt	TP53 mt
**10**		CDH1 mt, CDH1 Splice Site mt
**11**	CCND1 Amp+, EZH2 mt, FGF19 Amp+, FGF3 Amp+, FGF4 Amp+	
**12**		CDH1 Splice Site mt, CDKN2A mt, ARID1A mt, ARID2 mt
**13**	PIK3CA mt, ERBB3 mt, AXL mt, KDR mt	NF1 mt, ARID1A mt, CREBBP mt, CTCF mt, MLH1 mt
**14**		ARID1A mt
**15**	KRAS Amp+, CCNE1 Amp+, MDM4 Amp+	TP53 mt, FBXW7 mt, PTEN mt
**16**	HER2 Amp+	
**17**	KRAS mt	TP53 mt, CDKN2A mt
**18**		CDH1 Splice Site mt, LRP1B mt, MLL2 mt
**19**	Rictor Amp+	TP53 mt, KDM6A splice mt
**20**	CTNNB1	TP53 mt
**21**	HER2 mt, ERBB3 mt	TP53 mt (x2), DNMT3A mt, MSH2 mt

**Legend:** Amp+: Amplified, mt: mutation.

^**Т**^ Sequencing performed using Foundation One Platform (first generation – 186 genes), except patients 11–21, who were sequenced on the second generation platform (236 genes).

## Conclusions

GEC is one of the most common malignancies and second highest cause of cancer mortality worldwide. The extensive heterogeneity of etiology, patient ethnicity, tumor location, histology, and trial inclusion/exclusion criteria, have resulted in diverging treatment algorithms with lack of consensus worldwide. Trials are in motion in order to directly address these controversies, yet results will not be available for several years (ie ‘MAGIC vs CROSS Upper GI’ final data are expected ~2021). Ultimate treatment plans should be derived by multi-disciplinary review. Regardless, all current standard perioperative approaches improve absolute 5-yr survival for GEC patients ~10-15%, leaving much room for improvement. The promise of ‘personalized’ cancer care with therapies targeted toward specific molecular aberrations has great potential to significantly improve clinical outcomes. However, there is emerging understanding of the immense molecular heterogeneity within GEC (inter-patient heterogeneity), and even within an individual (intra-patient heterogeneity) (Table 3) [77]. This heterogeneity is a hurdle to advancing GEC treatment via targeted therapies. Current clinical trial design paradigms are challenged by heterogeneity, as they are unable to test targeted therapeutics against low frequency genomic aberrations with adequate power. Collaborative group multicenter and international studies with innovative designs and novel molecular diagnostic technologies will be necessary in order to accomplish these difficult but attainable goals [77,78].

## Abbreviations

GEC: Gastroesophageal cancer, including gastric, esophageal and esophagogastric adenocarcinoma; GC: Gastric cancer; EC: Esophageal adenocarcinoma (distal esophagus); EGJ: Esophagogastric junction; BE: Barrett’s esophagus, metaplasia; AC: Adenocarcinoma; SCC: Squamous cell cancer (of the proximal/mid esophagus); CRT: Chemoradiotherapy; RCT: Randomized controlled trial; mOS: Median overall survival; mPFS: Median progression free survival; mDFS: Median disease free survival.

## Competing interests

The authors declare that they have no competing interests.

## Authors' contributions

The outline was conceived by DC. Both authors contributed to initial drafts, edited versions, and the final version. Both authors read and approved the final manuscript.
